# A hybrid method of system dynamics and design of experiments for investigating the economic and environmental indicators of electricity industry

**DOI:** 10.1016/j.heliyon.2024.e31260

**Published:** 2024-05-17

**Authors:** Maryam Doroodi, Bakhtiar Ostadi, Ali Husseinzadeh Kashan, Seyed Hessameddin Zegordi

**Affiliations:** a Faculty of Industrial and Systems Engineering, Tarbiat Modares University, Tehran, Iran; b Faculty of Industrial and Systems Engineering, Tarbiat Modares University, Tehran, P.O. Box: 14115-111, Iran

**Keywords:** Electricity market, Export, CO2 emission, System dynamics, Design of experiments

## Abstract

Electricity plays a pivotal role in the socio-economic development of nations. However, heavy reliance on fossil fuels for electricity generation, as observed in Iran, poses significant environmental challenges. This study proposes a novel hybrid methodology that combines system dynamics modeling and Design of Experiments (DOE) to examine economic and environmental indicators within Iran's electricity sector. The system dynamics model delineates four key subsystems: consumption, production, CO2 emissions, and power trade. By integrating DOE into this framework, various economic and environmental metrics are assessed for the year 2040. Through a comprehensive analysis of variable impacts on these indicators, optimal levels are identified to achieve favorable outcomes. Notably, variables such as the allocation coefficient of export income to capacity development and electricity export price emerge as critical determinants. Due to economic, environmental, and economic-environmental indicators, the most appropriate level of allocation of export income towards capacity development is estimated at 30, 10, and 20 percent, respectively. The study recommends allocating 80 % of the capacity development budget to renewable energy sources and 20 % to thermal power plants to optimize future conditions. In business as usual, the Export CO2 emission damage to export income index will be 0.19. In implementing the proposed scenario, according to the economic-environmental index, this value will decrease and reach 1.73E-06, which indicates the improvement of electricity export from the economic-environmental dimension. This research underscores the importance of balancing economic prosperity with environmental sustainability in electricity industry planning and policy formulation.

## Highlights

• It is recommended to allocate only 20 % of the capacity development budget to thermal power plants.
• The export price has been introduced as the most important factor in influencing economic-environmental indicators.
• Regarding the indicators, the allocation coefficient of export income to capacity development and the electricity export price were the most effective variables.

## 1 Introduction

Electricity is the basis of the economic growth of any country. In all industries, including traditional and advanced industries, economic production is impossible without electricity [1]. However, it should be considered that in addition to economic growth, the resulting environmental impact should also be considered in planning and policy-making. Because one of the main sources of greenhouse gas emissions is the burning of fossil fuels to produce electricity. Among the greenhouse gases, CO2 has the greatest impact on climate change [2]. To slow climate change, many countries have focused on reducing CO2 emissions and moving toward sustainable development [3]. The CO2 emissions are also being forecasted in some studies [4,5]. The emissions of electricity generation were forecasted by Bakir et al. [6]. Although many innovations lead to the reduction of CO2 emissions resulting from the production of each kilowatt-hour of electricity, the net reduction of emissions from this sector is difficult to achieve [7]. Understanding the relationship between CO2 emissions and economic growth helps to formulate policies and develop resources in sustainable ways. There is a two-way relationship between CO2 emissions and economic growth. Usually, an increase in economic growth causes an increase in emissions, and controlling and reducing emissions will hurt the economy [8]. Therefore, precise planning and policies are needed in this field. However, it should be kept in mind that the planning and management of the electricity industry is a complex issue and the system dynamic (SD) has been introduced as a practical tool to solve this complexity [9,10]. This method, which has been used since 1970 to examine energy policies and their impact on energy-related issues [11]currently is very important in the long-term planning of the electricity system [12], and also a suitable method in the electricity market modeling [13]. In the following, a number of studies of the electricity industry are mentioned that have focused on the economic and environmental aspects with the help of the SD method.

In 2001, intending to evaluate the policy of encouraging the private sector to invest in the electricity industry in Pakistan, Qadratullah et al. presented an SD model. In this model, environmental limitations and available and limited resources were considered. The evaluation of this policy has been done in three dimensions including electricity supply, resource import dependency, and CO2 emissions. The results showed that the extension without changing the discussed policy effectively attracts the investment of electricity producers, but it is not without adverse consequences for the environment and the economy [14].

The government of Taiwan has planned policies regarding subsidies and tariffs to achieve the goal of reducing CO2 emissions. The goal of these policies is to attract companies and the general public to invest in and use photovoltaic systems. In 2012, HSU used a system dynamics approach to develop a simulation model to evaluate these policies. Using this model, policymakers can perform cost/benefit analyses for different combinations of existing policies, CO2 emission reduction targets, and budget constraints [3].

Until 2013, the Turkish government focused more on the security of supply and diversity of resources in the growing economy of Turkey and did not provide sufficient targets and appropriate laws regarding emissions. This year, Saysel and Hekimoğl presented an SD model to investigate the carbon emission issues of the Turkish electricity industry. The researchers concluded that with common policies related to carbon tariffs and subsidies, it is possible to achieve a 50 % reduction in carbon emissions compared to the business-*as*-usual scenario [15]. In the same year, the SD model was used to identify the dynamics of Canadian electricity production capacity. The researchers found that to achieve a stable electricity supply and demand system in Canada, in addition to traditional adjustment methods, significant new investments are needed for electricity production capacity and increasing productivity. The results showed that with the allocation of ten million dollars to the electricity sector from 2015 to 2025; Not only can Canada respond to the supply and demand gap, but it will also take one step towards a greener path in terms of the environment and emissions [16]. In 2014, Lopez et al. investigated changes in the energy matrix and GDP and its effect on CO2 emissions using the SD method. They stated that by focusing on the use of fossil fuels, even with a continuous increase in GDP, CO2 emissions can be controlled [17]. In China, the system dynamic method has been used to find the appropriate investment strategy for power companies concerning the impact on the carbon trading market (NCET) [18].

In 2022, Mostafaeipour et al. used SD modeling to investigate the impact of renewable energy development on CO2 emissions in Iran's electricity industry. Researchers studied economic, environmental, and technical subsystems. They investigated the impact of scenarios related to fuel tariffs and subsidies on CO2 emissions. This model showed that carbon emissions in 2040 can be reduced by 7–41 percent [19].

In 2022, Dehghan and Amin-Nasri modeled Iran's electricity industry, focusing on economic and environmental issues. They determined the optimal prices for the electricity sector and power plant fuels considering the goals of different stakeholders. The results showed that reducing the allocated budget and subsidy leads to an increase in exports, a decrease in emissions and water consumption, and an increase in the efficiency of thermal power plants and electricity supply [20]. Table 1 provides a summary of some related studies.

**Table 1:** Summary of some related articles.

References	Purpose	Key variables	Results
[21]	Determining a cost for carbon that leads to the economization of wind power production.	Carbon price - wind power production - total cost of production	Only when the price of carbon is significantly higher than the price of wind power production, wind power production is profitable for investors.
[22]	A study of electricity production from natural gas in China concerning subsidies and CO2 emission trading program in the Kyoto Protocol.	Electricity production - natural gas - subsidy - CO2 emission trading^a^	Subsidies play an important role in the development of electricity production from natural gas. Although the income from CO2 emission trading or green income is much lower than the income from selling electricity, it will lead to low carbon investments.
[23]	evaluating the impact of a combination of policies on PV installed capacity and reducing CO2 emissions	Photovoltaics (PV) – CO2 emissions - government costs	By increasing the capacity of PV, in addition to reducing CO2 emissions, in the optimal scenario, the cost of the government will be significantly reduced compared to the business-*as*-usual scenario
[24]	Analysis of the policies related to increasing the renewable energies penetration in electricity production and investigating how the policies affect the electricity system reliability.	Renewable energy - fluctuating electricity prices - security of supply - sustainable development	Renewable energies increase supply security, reduce price fluctuations, and stabilize the electricity industry.
[25]	Identifying the best fuel combination for electricity generation according to economic, social, political, technical, and environmental criteria and ranking the fuel combination for electricity generation according to 9 aspects of sustainable development in Zambia	An optimum combination of electricity generation and sustainable development	The best combination of Zambia's future electricity production includes hydro, wind, solar, and biomass, respectively.
[26]	Investigating the long-term economic effects and changes in electricity system costs under different scenarios of low-carbon electricity production in the UK.	Zero emission - carbon price - energy system cost	There is no win-win policy in this regard. However, by defining a suitable carbon price, it is possible to reduce the costs of the energy system in addition to achieving the goal of zero emissions.
[27]	Improving the sustainable development of China's electricity by considering the integrated effect of Green tradable certificate and carbon emission trading.	Carbon emission trading - Green tradable certificate - sustainable development - profit margin	Increasing investment in renewable electricity development technologies not only leads to a significant profit margin for electric companies but also improves access to sustainable development goals at the national level.
[28]	Simulation and study of the development process, growth rate, and carbon emission of thermal power plants	Development of thermal power plants - CO2 emission - economic development	There is a significant positive relationship between economic development and carbon emissions of thermal power plants
[29]	Examining the role of low-carbon or carbon-negative technologies, focusing on the social costs of carbon.	Energy mix in electricity production - CO2 emissions - Social cost of carbon	In a range of carbon social costs (0–500), the optimal energy mix includes 75 % of renewable energies. The social cost of carbon is very important in achieving the goal of carbon neutrality.
[19]	Studying economic-biological-environmental and technical dimensions of Iran's electricity industry to investigate the impact of renewable energy development on CO2 emissions.	CO2 emission - renewable energy development - fossil fuel tariff and subsidy	In addition to the fact that the development of renewable energy leads to a significant reduction in CO2 emissions; It is also affordable.
[30]	Investigating the possibility of carbon reduction goals concerning green and low-carbon investment in China during the period of Covid-19	Green investment - Co2 emission - Covid 19	Considering the impact of Covid-19, when government spending on the environment is small; The goal of carbon reduction will not be achieved in any of the examined scenarios. But regardless of the Pandemic, China will reach its commitments by 2030.

a According to the Kyoto Agreement, the CO2 emission capacity is defined for each country. Any country that does not fully use this capacity can sell its share to other countries.

Some authors have combined the SD method with other tools in the electricity industry studies. In 2007, Dimitrovski used the combination of the SD method with the optimization approach to simulate the mutual relationship between economic, technical, and bio-environmental factors [31]. In 2011, Pereira and Saraiva used the SD and genetic algorithms to solve the production planning problem in competitive electricity markets [32]. In 2011, Zhao et al. combined the SD and agent-based system model intending to evaluate policies related to solar power generation [33]. In 2019, Wang et al. combined the SD method and agent-based system model to study the behavior of all actors in the electricity market [34]. [25] To evaluate and identify the best fuel combination for electricity generation, using a multi-criteria decision-making framework based on hierarchical analysis and the SD method. Dehghan et al. have investigated the sustainable growth of Iran's electricity industry in the short and long term using the games theory in the system dynamic. They have also studied CO2 emissions, water, and gas consumption [35]. In addition, based on the study conducted by Ref. [13], the system dynamic modeling has been used simultaneously with methods such as generic algorithms, incorporating experimental economics, analytical hierarchy processes, iterative algorithms, decision trees, and game theoretical approaches [13].

It seems that the main motive for combining the system dynamic with other methods is to compress a significant amount of information into a single decision [9] which is very similar to the purpose of using the Design of Experiments (DOE) method. Although researchers have not combined SD and DOE in existing studies, they have used each method separately to examine the electricity market. Studying the economic and environmental aspects of the electricity market is one of the applications of these two methods [20,36,37]. examined the economic and environmental dimensions of the electricity market. Despite considerable progress in theory, the DOE is less widely applied in industry [38]. CO2 capture from power plants was studied using Taguchi DOE in 2019 [39]. To determine electricity prices and operating costs in Brazil, Queiroz et al., Applied DOE and neural networks [40].

SD modeling can provide the required input data and information for many statistical and mathematical methods and data-based decision-making, but the combination of this method with other tools has not been given much attention in studies of the electricity industry. According to the studies conducted in the literature, it seems that, in this field, there is a lack of combining the SD with the DOE method. To fill this gap in the literature, this research deals with the combination of the SD method and the DOE to study Iran's electricity industry. In this study, Iran's electricity industry has been modeled into four sub-systems using the SD method. The studied subsystems are electricity consumption, electricity production, emission, and trade. In fact, in this model, in addition to the economic dimension, the environmental dimension related to the electricity industry is focused on.

Like any country, some factors related to Iran's electricity industry do not have a specific value and have different levels. In this research, the export price, import price, carbon damage, the coefficient of export income allocation to power plant capacity development, and how to allocate the capacity development budget to Thermal and renewable power plants are the variables with different levels. The main goal of this article is to determine the most appropriate level of the mentioned variables to optimize environmental, economic, and economic-environmental indicators. Investigating and studying different situations caused by the combination of different levels related to these variables requires a large number of experiments, which is not optimal. For this purpose, the combined method of SD modeling with the Taguchi design of experiments has been used. In this regard, three categories of indicators, including economic, environmental, and economic-environmental indicators, have been extracted from the SD model and considered as the response variables for each proposed experiment in the Design of experiments. Finally, according to each of the investigated indicators, the most appropriate level of the mentioned factors has been determined. In addition, the rank of each factor about making changes in each of the indicators has also been determined. In the next section, the research methodology is introduced. Section three introduces the model and its validation. The fourth part is dedicated to the expression of the combined model and finally, the last two parts are the discussion, conclusion, and future studies.

## 2 Methodology

**Fig. 1:**
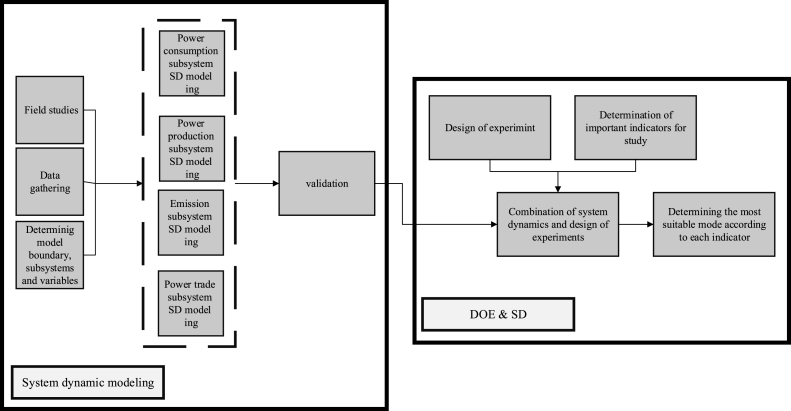
Research methodology.

The research methodology is illustrated in Fig. 1. A system dynamic model has been developed and validated after field studies, the definition of variables, and the establishment of model boundaries. Then, the indicators have been determined and the behavior of the indicators has been studied according to different levels of the existing variables with the help of combining the DOE method and SD modeling.

The data used in the model are collected from energy balance sheets published by the Ministry of Energy [41], domestic documents and articles of the electricity industry, and direct communication with electricity-related organizations and experts. According to the purpose of the study, the system boundary, as well as the available data, the variables in the model are determined Table A of the attachment contains a list of all variables. In this research, SD and DOE methods were implemented in Vensim and Mini-Tab 14 software, respectively.

## 3 Model

In this part, the general structure of the model, some causal loops, and the stock and flow (level and rate) diagrams are presented. The relevant equations are mentioned in Appendix (B).

### 3.1 Model structure

**Fig. 2:**
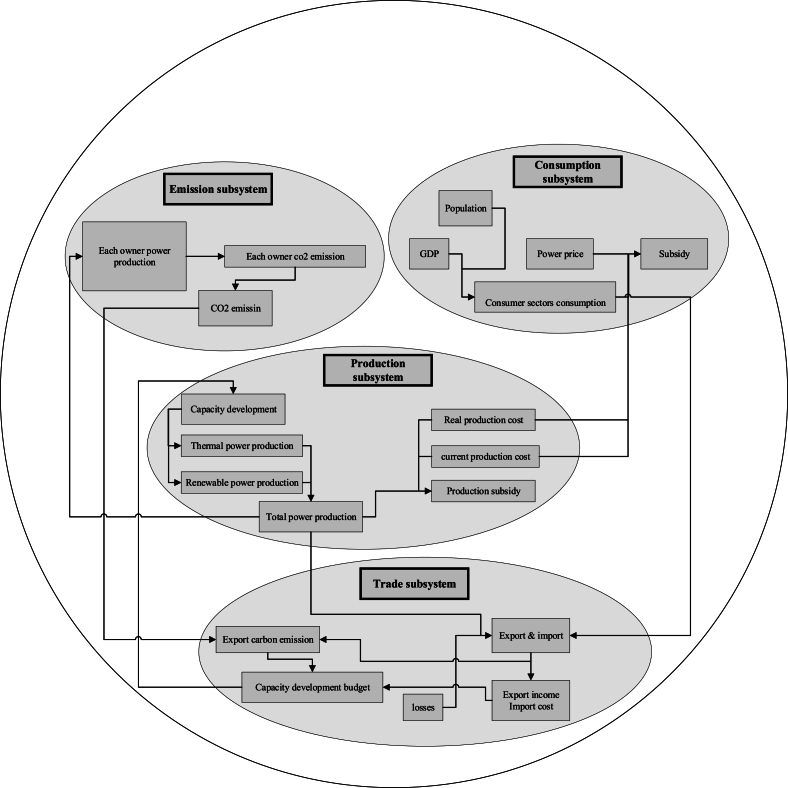
SD model structure.

Fig. 2 shows the structure, subsystems, and boundary of the model. In the consumption subsystem, according to the population and gross domestic product (GDP), electricity consumption is modeled in different consumer sectors. This subsystem's subsidy is then determined based on consumption and electricity prices in each sector. In the production subsystem, the production of thermal and renewable power plants is modeled. In addition to the consumer sector, subsidies are also allocated to the production sector In Iran, so the subsidy of the production subsystem is also calculated. Iran's power plants are under the ownership of three sectors, including large industries, the private sector, and the Ministry of Energy. In the emission subsystem, for more clarity and better decision-making, the ownership of power plants has been focused on. In the electricity trading sub-system, electricity imports and exports are modeled according to production, consumption, domestic consumption of power plants, and electricity losses. It is assumed that according to the export income, import cost, and the amount of carbon dioxide emission caused by electricity export, a budget is allocated to the capacity development of power plants, which is depicted in this subsystem.

**Fig. 3:**
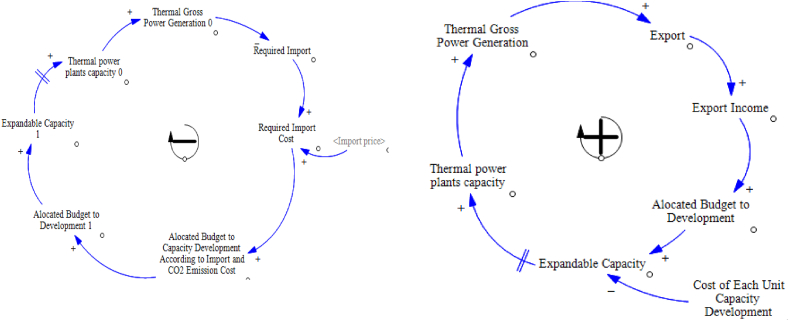
A reinforcement and balance loop of the SD model.

Fig. 3 shows a reinforcement and balance loop of the model. With the development of exports, the export income will increase, and by allocating a part of this income to capacity development, production will increase and then the export will increase again, and a reinforcement loop will be created. On the other hand, according to the required import cost, a budget is allocated for capacity development which leads to an increase in production. With the increase in production, the required import capacity and consequently the resulting cost will decrease and the budget allocated to capacity development will decrease and a balanced loop will be created.

### 3.2 Stock and flow diagram

**Fig. 4:**
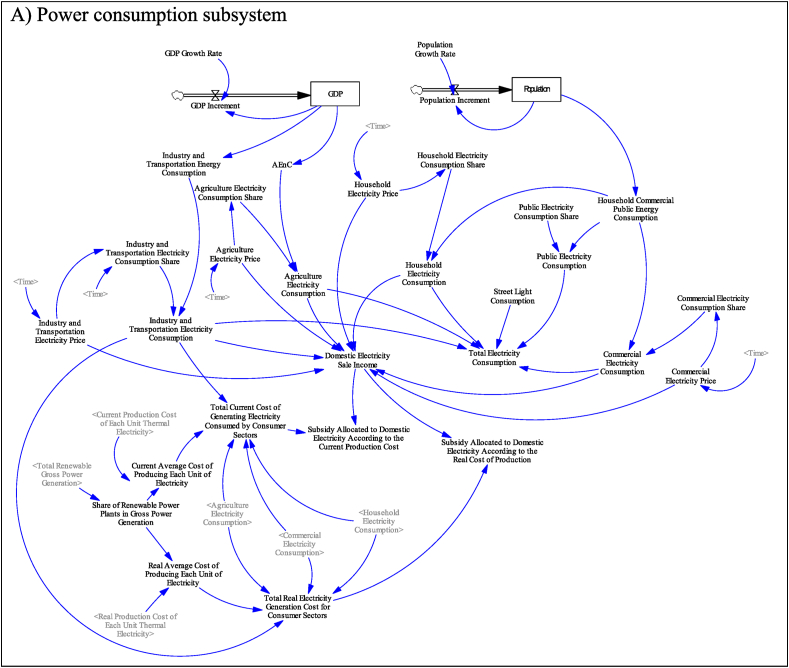

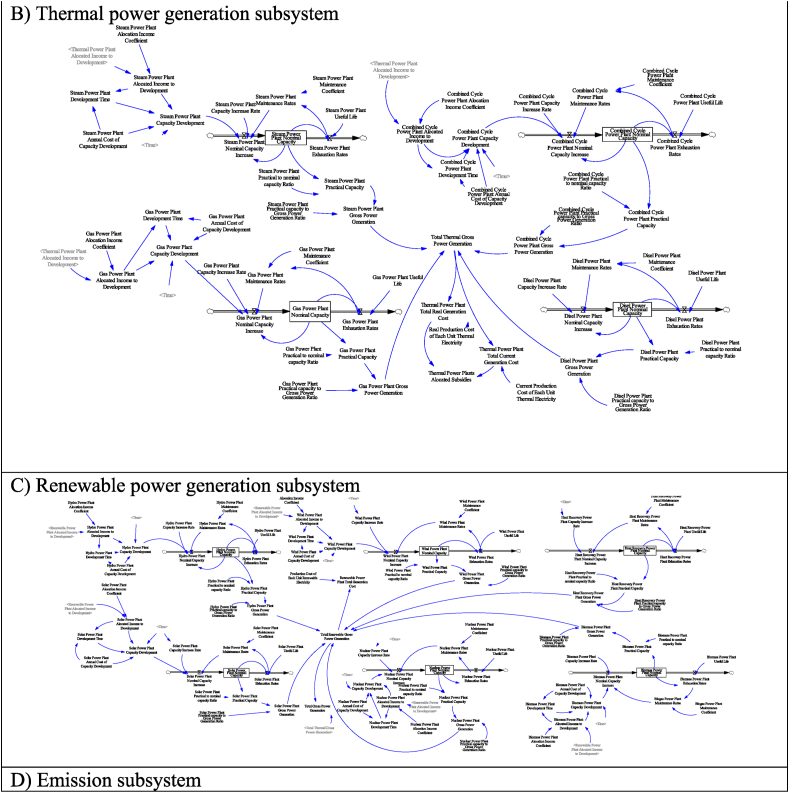

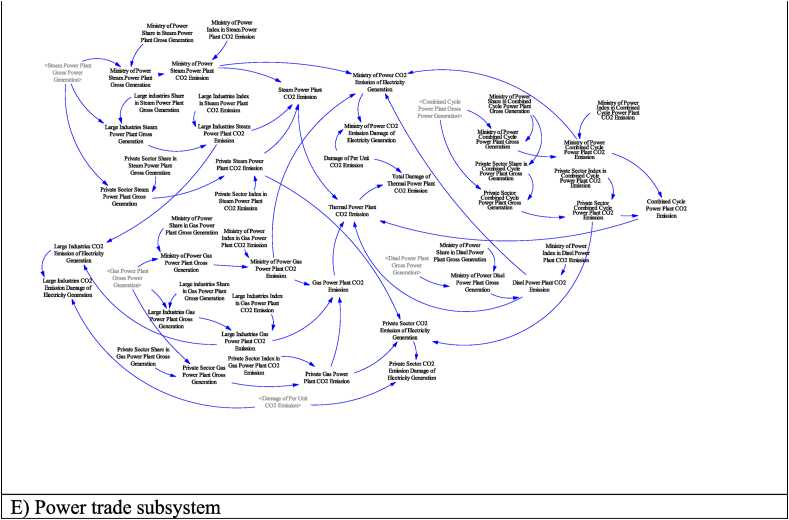

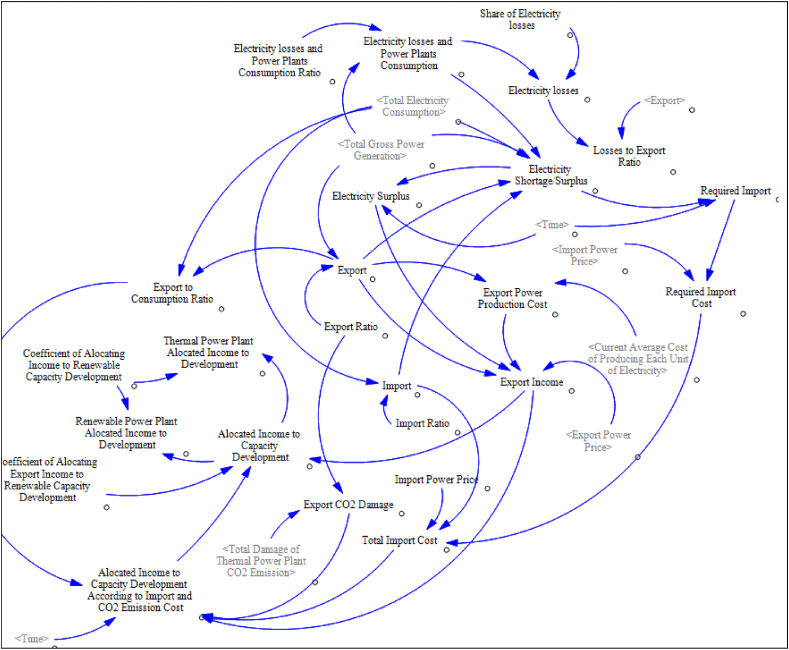
Stock and flow diagram of subsystems.

Fig. 4 shows the stock and flow diagram of the relevant subsystems. Diagram A is the electricity generation sub-system. In this subsystem, the electricity consumption of various consumer sectors, including household, public, commercial, agricultural, industry, and transportation, as well as the electricity required for street lighting, is modeled. The energy and electricity consumption of each consumer sector is modeled according to the population and GDP. In this subsystem, according to the sale price of electricity as well as the current and actual production price of electricity, current and actual electricity subsidies are simulated. In calculating the current electricity subsidy, the subsidized price of fuel for power production is taken into account, and in the actual subsidy, the actual price of fuel is considered to calculate the actual cost price.

Diagrams B and C show electricity production in thermal and renewable power plants, respectively. Thermal power plants include gas, steam, combined cycle, and diesel power plants. Renewable power plants include wind, solar, hydropower, biogas, heat recovery, and nuclear recycling. In each power plant, the gross power generation is calculated according to the nominal and practical capacity. Capacity development due to budget allocation is also included in the model for each power plant. In addition to the consumption sector, subsidies are also given to the electricity production sector, and these subsidies are considered in the model.

Diagram D is the emission subsystem. In this subsystem, CO2 emission caused by electricity production in thermal power plants is modeled. The ownership of power plants, which in Iran is the responsibility of three sectors including the private sector, large industries, and the Ministry of Energy, has been considered in this subsystem. This approach has been adopted for ownership-oriented prediction, decision, and policy-making.

Diagram E shows the electricity trading subsystem. Import, export, cost, and income related to them are modeled in this part. It is assumed that a budget will be allocated to the capacity development of power plants according to the export income, the required import, and the carbon damage caused by electricity export. this part is also included in the trading subsystem.

The equations of the SD model, along with the corresponding table of abbreviations, are in the appendix.

### 3.3 Validation

The outputs of the model have been compared with historical data for model validation. In the following, in each subsystem, for the sake of brevity, the graph and the MAPE error related to only one variable are presented. As shown in Table 2, the simulation has suitable accuracy.

**Table 2:** Validation table.

subsystem	Consumption	Production
Simulated versus historical data		
MAPE	4.4	3.26
subsystem	Emission	Trade
Simulated versus historical data		
MAPE	5.3	2.56

## 4 Combination of system dynamics and design of experiments

Electricity Production and export, along with the economic advantage, lead to CO2 emission. So, the economic benefit of this issue should be examined and taken into consideration along with its environmental damage. For this purpose, economic, environmental, and economic-environmental indicators can be studied with the help of the SD model presented in this research. On the other hand, the value of some parameters in the field of electricity has several levels. The multi-level variables in this research include export price, import cost, emission damage per unit of carbon dioxide, allocation coefficient of export income to capacity development, and also how to allocate income to thermal and renewable power plants. According to experts, the price of exported electricity is defined as between 6 and 11 cents, and in some cases, it is five times the price of domestic electricity. The import cost is 3–5 cents. The cost of each ton of carbon dioxide emissions has been announced as 3, 10, or 80 dollars by Product Carbon Footprint (PCF), the result of a picture of prices in the carbon future market, Intergovernmental Panel on Climate Change (IPCC) respectively. The allocation coefficient of export income to capacity development is considered to be 10 %–30 %. On the other hand, how to allocate income to capacity development of renewable and thermal power plants can vary between 0 and 100 percent for each power plant. Table 3 shows the factors and their related levels.

**Table 3:** Defined Factors and related levels in the DOE.

Factor level	Export price (cents/kWh)	Import cost (cents/kWh)	Emission damage ($/ton CO2)	Allocation coefficient of export income to capacity development (percentage)	How to allocate income to capacity development (percentage)
1	6	3	3	10	50–50
2	8	3.5	10	15	60–40
3	10	4	80	20	40–60
4	11	4.5	The average of the first three cases (31)	25	20–80
5	5 times the average domestic prices	5	Average of the last two (45)	30	80–20

The number of states resulting from the combination of different levels of mentioned multilevel factors is very large and it is not possible to evaluate all of them. Therefore, to solve this problem, the method of Taguchi design of experiments (TDOE) is used. In this method, 5 levels (according to Table 3) are defined for each variable or factor, and the TDOE method suggested 25 experiments (Table 4). Without using the TDOE, it is necessary to perform 3125 (the number of levels to the power of the number of factors) tests.

**Table 4:** Experiments suggested by TDOE.

The number of suggested test	Factors				
	Export price (cents/kWh)	Import cost (cents/kWh)	Emission damage ($/ton CO2)	Allocation coefficient of export income to capacity development (percentage)	How to allocate income to capacity development (percentage)
1	6	3	3	10	50–50
2	6	3.5	10	15	60-40^a^
3	6	4	80	20	40–60
4	6	4.5	31	25	20–80
5	6	5	45	30	80–20
6	8	3	10	25	40–60
7	8	3.5	80	30	20–80
8	8	4	31	10	80–20
9	8	4.5	45	15	50–50
10	8	5	3	20	60–40
11	10	3	80	15	80–20
12	10	3.5	31	20	50–50
13	10	4	45	25	60–40
14	10	4.5	3	30	40–60
15	10	5	10	10	20–80
16	11	3	31	30	60–40
17	11	3.5	45	10	40–60
18	11	4	3	15	20–80
19	11	4.5	10	20	80–20
20	11	5	80	25	50–50
21	average	3	45	20	20–80
22	average	3.5	3	25	80–20
23	average	4	10	30	50–50
24	average	4.5	80	10	60–40
25	average	5	31	15	40–60
BAU^b^	8.5	4	31	–	–

a The left number is the percentage of revenue allocation to the thermal power plant and the right number is the percentage of revenue allocation to the renewable power plant.

b Business As Usual.

In conducting the proposed experiments, three categories of indicators have been considered as response variables, economic, environmental, and economic-environmental indicators. To perform each experiment, Iran's electricity SD model has been used. Each of the experiments proposed by TDOE was implemented in the SD model and the value of the mentioned indicators was extracted from the SD and Entered into the TDOE as the response variable for further analysis. Because the purpose of this research is to predict the most appropriate levels of factors in 2040, the values related to 2040 have been extracted from the SD model and considered as the values of response variables in TDOE analysis. For example, in test number one (in Table 4) proposed by TDOE, export price, import cost, and carbon damage are considered as 6, 6, and 3 respectively. Also, the allocation coefficient of export income to capacity development is 20 % and the method of budget allocation is 50-50 (50 % of the budget considered for capacity development should be allocated to the thermal power plant and 50 % to the renewable power plant). These proposed levels are set in the SD model and the mentioned response variables are extracted and entered into TDOE for further analysis. It should be noted that in the implementation of all proposed experiments in the SD model, the budget allocated to thermal power plants is equally divided between steam, gas, and combined power plants and the budget allocated to renewable power plants is also equally divided between the hydro, solar, wind, nuclear and biogas power plants.

### 4.1 Determination of optimal conditions based on economic indicators with the combined method of the TDOE and SD model

In this part, gross power production and export income are examined as economic indicators A and B, respectively. The 25 experiments suggested by the TDOE were implemented in the SD model and the predicted values of 2040 related to these two indicators were extracted from the SD and entered as the response variables of TDOE. The estimated values of these two indicators can be seen in the second and third columns of Table 10.

#### 4.1.1 The output of TDOE related to the gross power production (economic index A)

As it comes from the definition of this indicator, the higher the value, the more suitable the conditions are, therefore, in TDOE analysis, the larger is better option has been selected for the response variable. Table 5 shows that the allocation coefficient of export income to capacity development with a rank of 1 will have the greatest impact on the investigated response variable (gross domestic production) in 2040. The export price, how to allocate income to the thermal and renewable power plants capacity development, the import price, and finally the carbon damage have been ranked 2nd to 5th respectively. Diagram 5 shows at what level each variable will create the optimal state for the response variable.

**Table 5:** The output of the TDOE regarding the economic response variable A.

Allocate					
Revenue					
to Export income					
	Export	Import	Emission	capacity	Coefficient
Level	Price	Price	damage	de	alloc
1	224.3	245.0	241.4	243.8	219.4
2	236.5	241.3	242.5	241.0	234.9
3	242.3	243.9	243.1	243.1	246.2
4	245.5	241.3	243.9	245.4	250.8
5	264.4	241.5	242.3	239.8	261.9
Delta	40.1	3.7	2.5	5.7	42.5
Rank	2	4	5	3	1

**Fig. 5:**
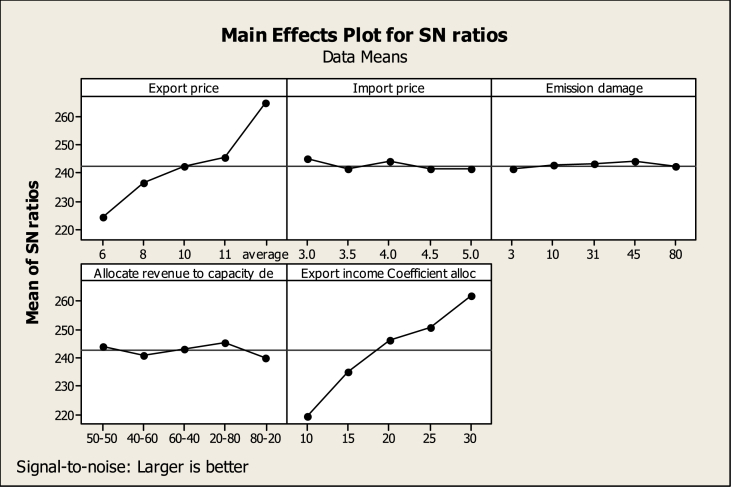
Main effects plot for SNR, according to economic index A (TDOE output).

According to Fig. 5, the export price variable at level 5 (the highest point in the SNR diagram), or five times the average domestic prices will lead to the highest gross power production in 2040.

The import price variable should be at the level of one or three cents per kilowatt hour and the carbon damage variable should be at the level of 4 or 45 cents per ton of CO2 emissions. The allocation of the budget to thermal and renewable power plants should be 80-20 according to diagram 5 (20 % of the budget should be allocated to the development of thermal power plants and 80 % to the development of renewable power plants). Finally, the SNR diagram related to the allocation coefficient of export income shows that by allocating 30 % of export income to capacity development the most suitable conditions will be created in 2040 for gross power production.

#### 4.1.2 The output of TDOE related to the export income (economic index B)

Similar to index A, the higher the value, the more suitable the conditions are, therefore, in TDOE, the larger is better option has been selected for the response variable. According to Table 6, the export price with the first rank will be the most effective factor in the response variable changes and the carbon damage has also shown the least impact (rank 5).

**Table 6:** The output of the TDOE regarding the economic response variable B.

Allocate					
Revenue					
to Export income					
	Export	Import	Emission	capacity	Coefficient
Level	Price	Price	damage	de	alloc
1	198.6	226.2	222.5	225.0	200.5
2	213.2	222.5	223.7	222.2	216.1
3	221.0	225.1	224.3	224.3	227.4
4	225.0	222.5	225.0	226.6	232.0
5	261.1	222.7	223.5	221.0	243.0
Delta	62.5	3.7	2.5	5.7	42.5
Rank	1	4	5	3	2

**Fig. 6:**
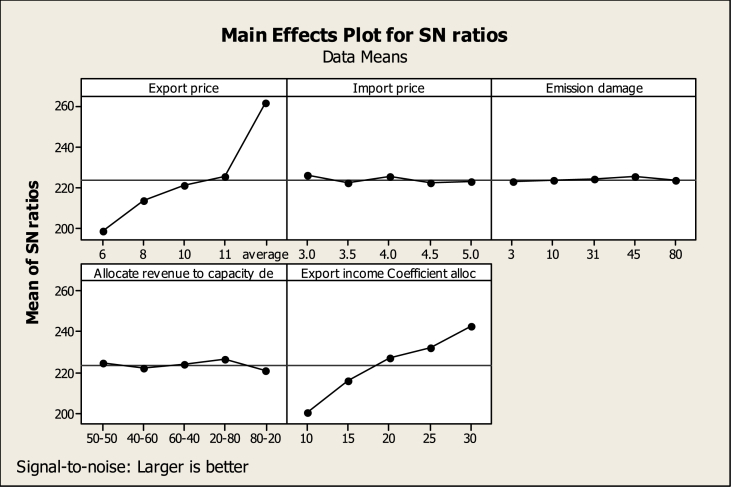
Main effects plot for SNR, according to economic index B (TDOE output).

**Fig. 7:**
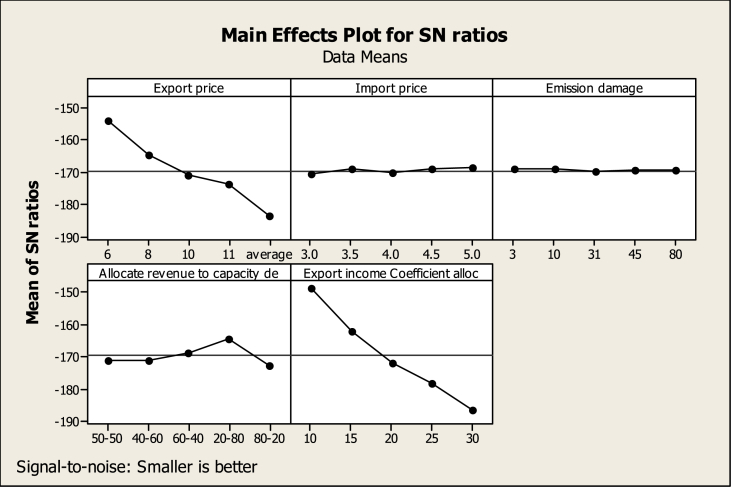
Main effects plot for SNR, according to environmental index A (TDOE output).

According to the SNR diagram (Fig. 6), the variables of export price, import price, and carbon damage will lead to the highest export income in 2040 at level 5, level 1, and level 4, respectively In addition, the SNR chart shows that to reach the most appropriate response variable conditions, 30 % of export income must be allocated to capacity development. While 20 % of this budget should be allocated to thermal power plants and 80 % to renewable power plants (see Fig. 7).

### 4.2 Determining optimal conditions according to environmental indicators using the combined method of TDOE and SD model

The CO2 emission due to electricity production in thermal power plants has been investigated as environmental response indicator A. In addition, because electricity production in the private sector in Iran emits the most CO2; This index has also been evaluated as environmental indicator B. The lower the value of both indicators, the more appropriate conditions are. Therefore, in TDOE, the “smaller is better” option is chosen for them.

#### 4.2.1 The output of TDOE related to the thermal power plant's CO2 emission (environmental index A)

According to Table 7, the allocation coefficient of export income to capacity development has the highest rank in creating changes in the response variable (CO2 emissions of thermal power plants). Export price, how to allocate budget to thermal and renewable power plants, import price, and finally, carbon damage are ranked second to fifth. Regarding the determination of the most suitable level of each factor, according to diagram 7, it can be said that the export price at the level of one or 6 cents per kilowatt hour will lead to the lowest amount of CO2 emissions from thermal power plants in 2040. In this situation, the import cost and carbon damage should be at the level of 5 cents per kilowatt hour and 3 cents per ton, respectively. In addition, only 10 % of the export income must be allocated to the power plant's capacity development. While 20 % of this budget should be allocated to thermal power plant development and 80 % to renewable power plant development. By adopting these conditions, the lowest amount of carbon dioxide from electricity production in thermal power plants will be emitted in 2040.

**Table 7:** The output of the TDOE regarding the environmental response variable A.

Allocate Export income					
	Export	Import	Emission	revenue to	Coefficient
Level	Price	Price	damage	capacity de	alloc
1	−154.2	−170.6	−169.3	−171.0	−148.7
2	−165.0	−169.2	−169.2	−171.1	−162.2
3	−170.9	−170.2	−169.8	−168.7	−171.9
4	−173.8	−169.0	−169.7	−164.2	−178.2
5	−183.7	−168.6	−169.7	−172.6	−186.6
Delta	29.6	1.9	0.5	8.4	37.9
Rank	2	4	5	3	1

#### 4.2.2 The output of TDOE related to the private sector CO2 emission duo to power production (environmental index B)

According to Table 8, the allocation coefficient of export income to power plant capacity development will rank first in influencing CO2 emissions caused by private sector electricity production. The export price will have the second rank in causing changes in the response variable in 2040. How to allocate income to thermal and renewable power plants, carbon damage and import prices will rank third to fifth respectively. Diagram 8 shows the best level of each variable to create the most suitable conditions for the response variable in 2040. If each variable adopts the level corresponding to the highest point in the SNR diagram, the response variable will have the most suitable conditions.

**Table 8:** The output of the TDOE regarding the environmental response variable B.

Allocate Export income					
	Export	Import	Emission	revenue to	Coefficient
Level	Price	Price	damage	capacity de	alloc
1	−270.1	−286.4	−285.1	−286.8	−264.6
2	−280.9	−285.1	−285.1	−287.0	−278.1
3	−286.8	−286.1	−285.7	−284.6	−287.8
4	−289.8	−284.9	−285.6	−280.1	−294.1
5	−299.5	−284.5	−285.6	−288.5	−302.5
Delta	29.4	1.9	0.6	8.4	37.9
Rank	2	4	5	3	1

### 4.3 Determining optimal conditions according to economic-environmental indicators using the combined method of TDOE and SD model

**Fig. 8:**
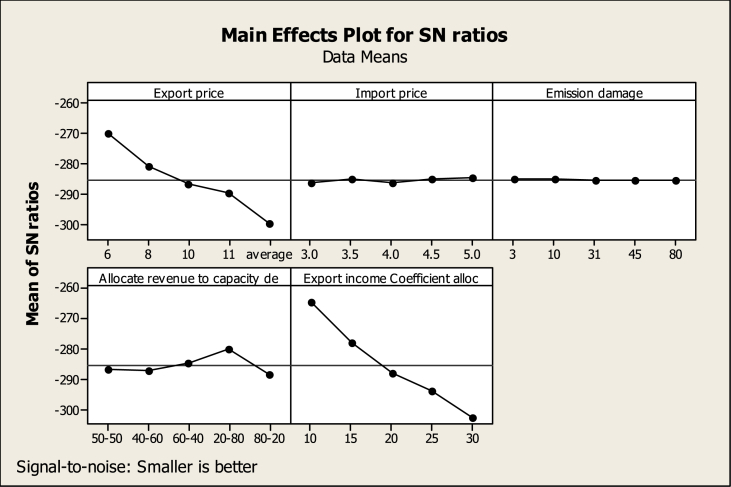
Main effects plot for SNR, according to environmental index B (TDOE output).

The ratio of export electricity production cost and CO2 emission damage caused by export to export income has been investigated as an economic-environmental index (see Fig. 8).

#### 4.3.1 The output of TDOE related to the economic-environmental response variable

**Fig. 9:**
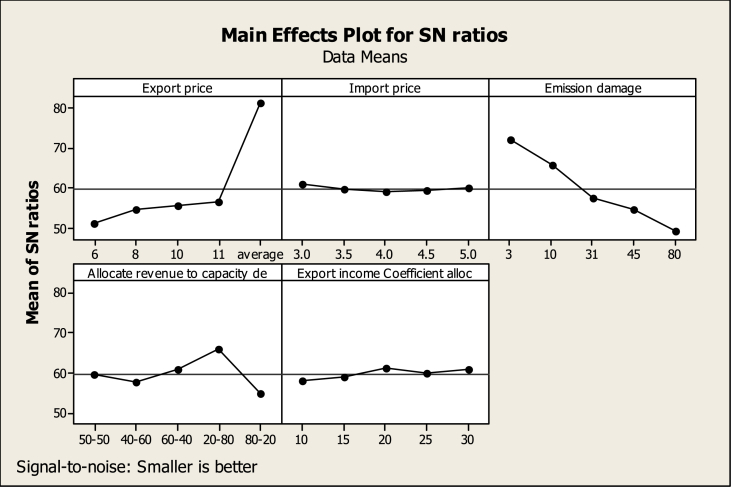
Main effects plot for SNR, according to economic-environmental index (TDOE output).

The influence rank of each variable on the economic-environmental index is available in Table 9. According to this table, the electricity export price, the CO2 emission damage, the way of allocating income to thermal and renewable power plants, the allocation coefficient of export income to capacity development, and finally the import price is ranked first to fifth respectively. In other words, with a change in the electricity export price, the largest effect in the response variable will be observed. According to Fig. 9, the response variable will have the lowest level at level five related to the export price, five times the average domestic prices. At the same time, the variables of import price, emission damage, the method of allocating budget to power plants, and the allocation coefficient of export income to capacity development must adopt levels 1, 1, 4, and 3, respectively.

**Table 9:** The output of the TDOE regarding the economic-environmental response variable.

Allocate					
Revenue					
to Export income					
	Export	Import	Emission	capacity	Coefficient
Level	Price	Price	damage	de	alloc
1	51.02	60.99	72.08	59.51	58.16
2	54.63	59.60	65.46	57.79	58.88
3	55.60	59.07	57.49	60.73	61.07
4	56.57	59.18	54.63	65.82	59.76
5	81.00	59.97	49.15	54.96	60.93
Delta	29.98	1.91	22.93	10.86	2.90
Rank	1	5	2	3	4

**Table 10:** The 2040 estimated values of each indicator in each proposed experiment of the TDO. Extracted from the SD model.

Test number	Economic indicators		Environmental indicators		Economic-environmental indicator
	Total gross power production	Export income	Thermal power plant's CO2 emission	Private sector CO2 emission	The ratio of export electricity production cost and CO2 emission damage caused by export to export income
1	1.59E+10	8.2023e+08	6.25E+06	3.89E+12	6.45E-04
2	7.65E+10	3.95565e+09	3.17E+07	1.98E+13	1.81E-03
3	1.99E+11	1.02597e+10	5.53E+07	3.45E+13	9.05E-03
4	5.42E+11	2.80007e+10	7.21E+07	4.50E+13	1.93E-03
5	9.29E+11	4.80145e+10	4.44E+08	2.79E+14	8.65E-03
6	1.97E+12	1.35911e+11	4.42E+08	2.76E+14	8.58E-04
7	7.46E+12	5.1395e+11	6.98E+08	4.36E+14	2.46E-03
8	6.55E+10	4.51167e+09	3.33E+07	2.09E+13	4.78E-03
9	2.09E+11	1.44103e+10	7.04E+07	4.39E+13	4.65E-03
10	6.52E+11	4.49485e+10	2.40E+08	1.50E+14	4.70E-04
11	4.97E+11	4.27968e+10	2.42E+08	1.52E+14	9.34E-03
12	1.76E+12	1.51351e+11	5.00E+08	3.12E+14	2.23E-03
13	4.01E+12	3.4531e+11	1.30E+09	8.11E+14	3.59E-03
14	1.52E+13	6.23367e+09	2.73E+09	1.70E+15	2.95E-04
15	7.24E+10	1.28072e+12	1.23E+07	7.67E+12	5.73E-04
16	1.35E+13	1.30634e+10	3.97E+09	2.48E+15	2.09E-03
17	1.38E+11	8.53157e+10	3.96E+07	2.47E+13	2.91E-03
18	9.01E+11	1.77472e+11	1.12E+08	7.01E+13	2.46E-04
19	1.87E+12	7.15565e+11	8.73E+08	5.48E+14	1.09E-03
20	7.55E+12	5.84224e+13	1.88E+09	1.17E+15	4.44E-03
21	8.54E+13	1.05088e+13	1.64E+09	1.01E+15	5.25E-05
22	1.54E+13	1.39482e+14	4.48E+09	2.78E+15	4.34E-05
23	2.04E+14	4.54346e+11	1.35E+10	8.24E+15	4.47E-05
24	6.64E+11	4.95457e+12	1.50E+08	9.32E+13	5.60E-04
25	7.25E+12	5.84224e+13	5.80E+08	3.58E+14	9.87E-05
BAU	862835	1495.74	448.383	3.12274e+08	6.45E-04

It can be said that in the future, the increase in export prices will lead to an increase in income, the budget allocated to capacity development, and an increase in CO2 emissions. However, by allocating more budget to renewable power plants, the increase in power production and export exceeds the growth of CO2 emission and leads to the improvement of the index. In other words, the growth of carbon damage will be lower than the growth of export income. Although with the increase in import cost in the same proportion (and according to the ratio of export to production), a budget is allocated to capacity development, the outputs show that the most suitable import price is the lowest level, in other words, at this level of capacity development, the growth of export income will be higher than the growth of carbon dioxide emission. Regarding how to allocate a budget to the development of thermal and renewable power plants, it can be said that by allocating equal budgets between two types of power plants, power production and exports will increase. However the growth of carbon emission and production cost will be more than the growth of production and export income and will lead to the increase of the index. By allocating more funds to renewable power plants, compared to thermal power plants, the index will have more favorable conditions. By allocating 80 % of income to renewable power plants, along with production growth, CO2 emission is also controlled. It seems that the conditions will improve in the future by increasing the allocation coefficient of export income to capacity development along with more focus on the renewable power plant's capacity development. but the output of the TDOE states that the allocation of only 20 % of the export income to capacity development will lead to the most appropriate situation of the economic-environmental index. These results show the importance of CO2 emission control along with power production and export growth.

## 5 Discussion and conclusion

Table 11 presents the rank of each variable in influencing the indicators. the allocation coefficient of export income to capacity development will cause the most changes in environmental indicators because it took the first rank in both environmental indicators. While focusing on the economic-environmental indicator, the first rank of making changes was related to the export price. According to the economic indexes A and B, respectively, the allocation coefficient of export income to capacity development and export price has shown the greatest impact on the response variable changes.

**Table 11:** The rank of each factor in influencing the indicators.

Factors	Economic indicators		Environmental indicators		Economic-environmental indicator
	Gross power production	Export income	Thermal power plant's CO2 emission	Private sector CO2 emission	The ratio of export electricity production cost and CO2 emission damage caused by export to export income
Export price	2	1	2	2	1
Import price	4	4	4	4	5
Emission damage	5	5	5	5	2
Allocate revenue to capacity development	3	3	3	3	3
allocation coefficient of Export income	1	2	1	1	4

Table 12 shows the most appropriate level of each variable according to each of the indicators. The export price, according to the economic and economic-environmental indicators, at the level of 5 times the average domestic prices, and according to the environmental indicators, at the level of 6 cents per kilowatt hour, lead to the best conditions of the response variables. The Import price according to economic and economic-environmental indicators at the level of 6 cents per kilowatt hour and according to environmental indicators at the level of 5 cents per kilowatt hour lead to the best conditions of response variables. The carbon damage variable focusing on environmental and economic-environmental indicators is proposed at the level of 3 cents per ton of emission and 45 cents according to economic indicators. The allocation coefficient of export income to capacity development, according to economic, environmental, and economic-environmental indicators, is 30, 10, and 20 %, respectively. Finally, according to all the indicators, by allocating 20 % (Allocation coefficient) of the capacity development budget to the thermal power plants and 80 % to renewable power plants, the response variables will give the most appropriate conditions in 2040.

**Table 12:** The most appropriate level of each factor for indicators.

Factors	Economic indicators		Environmental indicators		Economic-environmental indicator
	Gross power production	Export income	Thermal power plant's CO2 emission	Private sector CO2 emission	The ratio of export electricity production cost and CO2 emission damage caused by export to export income
Export price	Average	average	6	6	average
Import price	3	3	5	5	3
Emission damage	45	45	3	3	3
Allocate revenue to capacity development	20–80	20–80	20–80	20–80	20–80
allocation coefficient of Export income	30	30	10	10	20

### 5.1 5-1 comparison of the SD results of the proposed model by TDOE (according to the economic-environmental indicator) and BAU

In this section, the SD model has been implemented by adjusting the parameters according to the suggested levels by the TDOE method in the economic-environmental index. Table 13 presents the SD results of the proposed model in comparison to the BAU scenario in 2040 for some important variables. In the SD BAU scenario, the average levels are considered for the multilevel variables (for example the power export price is 8.5, i.e. the middle of the range 6–11). As it is clear in Table 13, due to the increase in exports and the resulting income in the TDOE suggested model, the ratio of the emission damage to export income in the BAU scenario has a more inappropriate value.

**Table 13:** Comparison of the SD results of the proposed model by TDOE (according to economic-environmental indicator) and BAU.

Variable/indicator	SD output according to BAU	SD output according to TDOE proposed model
Export CO2 emission damage to export income	0.190068	1.73E-06
Thermal power production subsidies to export income	35.2	2.92E-03
Electricity shortage (million KWH)	820275	0

The increase in power production capacity leads to an increase in government subsidies to the electricity sector, so the ratio of subsidies to export income has also been examined. As it is clear from the definition of this index, its reduction will be desirable for the government (from the point of view of the consumers and the producers, it should be investigated). According to the obtained results, if the current trend continues (BAU), the ratio of thermal power plant production subsidies to export income will be higher than the suggested model by the TDOE.

Due to the necessity of implementing government obligations in response to internal power needs as well as international contracts, the lack of electricity has also been investigated in this section, and this variable is reduced to zero in 2040 in the proposed model by TDOE. In general, it can be said that the proposed model by the TDOE will lead to a more favorable situation for the future of the electricity sector.

## 6 Conclusion

The electricity industry has many complexities and the system dynamic method is used to solve this complexity. On the other hand, for some variables in the electricity industry, different levels can be considered, and studying all the combinations of these variables and their corresponding values is a tedious task. Therefore, in this study, the combined method of system dynamics and DOE was used to study the Iranian electricity industry. In this regard, three categories of indicators including economic, environmental, and economic-environmental indicators have been studied. Finally, the best levels of the mentioned variables are presented to determine the best conditions of the indicators in 2040.

The collection of data from organizations is one of the limitations of this study. A further challenge was posed by the COVID-19 pandemic, which made conducting site visits and obtaining information from these companies extremely difficult, if not impossible. Furthermore, some data were deemed confidential and inaccessible, such as electricity export data. Despite these constraints, the model boundaries were adopted. In this study, it is assumed that Iran's political relations with other countries will allow it to continue to trade electricity. Also, As no plan has been defined for the development of diesel power plants in Iran in the future, this research did not examine the development of this power plant.

### 6.1 Future work

In the future, it is possible to study social aspects also indicators related to the different stakeholders' points of view by the existing method. In addition, the model can be adapted to examine the impact of external factors (e.g., geopolitical developments, technological advancements, etc.) on key variables. Using the SD method in conjunction with other optimization techniques such as Generalized Pattern Search and Particle Swarm Analysis and comparing the results with the SD and DOE combination can be investigated in the future.

This research did not receive any specific grant from funding agencies in the public, commercial, or not-for-profit sectors.

## Data availability statement

The data associated with this study have not been deposited in a repository that is publicly accessible but will be made available upon request.

## CRediT authorship contribution statement

**Maryam Doroodi:** Writing – original draft, Validation, Software, Formal analysis, Data curation. **Bakhtiar Ostadi:** Writing – review & editing, Writing – original draft, Validation, Supervision, Methodology, Investigation, Conceptualization. **Ali Husseinzadeh Kashan:** Validation, Investigation. **Seyed Hessameddin Zegordi:** Validation, Investigation.

## Declaration of Competing interest

We wish to confirm that there are no known conflicts of interest associated with this publication and there has been no significant financial support for this work that could have influenced its outcome.

We confirm that the manuscript has been read and approved by all named authors and that there are no other persons who satisfied the criteria for authorship but are not listed. We further confirm that the order of authors listed in the manuscript has been approved by all of us.

We confirm that we have given due consideration to the protection of intellectual property associated with this work and that there are no impediments to publication, including the timing of publication, with respect to intellectual property. In so doing we confirm that we have followed the regulations of our institutions concerning intellectual property.

We understand that the Corresponding Author is the sole contact for the Editorial process (including Editorial Manager and direct communications with the office). He is responsible for communicating with the other authors about progress, submissions of revisions and final approval of proofs. We confirm that we have provided a current, correct email address which is accessible by the Corresponding Author and which has been configured to accept email from (bostadi@modares.ac.ir)

## Figures and Tables

**Table:** 

**Variable Name**	Abbreviation	Variable Name	Abbreviation
Thermal Power Plants Alocated Subsidies	TPPAS	Household Commercial Public Energy Consumption	HCPEnC
Thermal Power Plant Total Real Generation Cost	TPPTRGC	Population	Pop
Thermal Power Plant Total Current Generation Cost	TPPTCGC	Agriculture Energy Consumption	AEnC
Total Renewable Gross Power Generation	TRGPG	Gross Domestic Product	GDP
Wind Power Plant Gross Power Generation	WPPGPG	Industry and Transportation Energy Consumption	ITEnC
Heat Recovery Power Plant Gross Power Generation	HRPPGPG	Commercial Electricity Price	CEP
Hydro Power Plant Gross Power Generation	HPPGPG	Household Electricity Price	HEP
Biomass Power Plant Gross Power Generation	BPPGPG	Agriculture Electricity Price	AEP
Solar Power Plant Gross Power Generation	SOPPGPG	Industry and Transportation Electricity Price	ITEP
Nuclear Power Plant Gross Power Generation	NPPGPG	GDP Increment	GDPI
Renewable Power Plant Total Generation Cost	RPPTGC	Population Increment	PopI
Total Gross Power Generation	TGPG	Agriculture Electricity Consumption Share	AECS
Ministry of Power Share in Gas Power Plant Gross Generation	MOPSGPPGG	conversion factor	CF
Ministry of Power Gas Power Plant Gross Generation	MOPGPPGG	Agriculture Energy Consumption	AEnC
Large Industries Gas Power Plant Gross Generation	LIGPPGG	Total Electricity Consumption	TEC
Large Industries Share in Gas Power Plant Gross Generation	LISGPPGG	Street Light Consumption	SLC
Private Sector Share in Gas Power Plant Gross Generation	PSSGPPGG	Industry and Transportation Electricity Consumption	ITEC
Private Sector Index in Gas Power Plant CO2 Emission	PSIGPPCE	Agriculture Electricity Consumption	AEC
Ministry of Power Index in Gas Power Plant CO2 Emission	MOPIGPPCE	Commercial Electricity Consumption	CEC
Large Industries Index in Gas Power Plant CO2 Emission	LIIGPPCE	Household Electricity Consumption	HEC
Private Sector Gas Power Plant Gross Generation	PSGPPGG	Public Electricity Consumption	PEC
Private Sector Index in Gas Power Plant CO2 Emission	PSIGPPCE	Total Real Electricity Generation Cost for Consumer Sectors	TREGCCS
Gas Power Plant CO2 Emission	GPPCE	Real Average Cost of Producing Each Unit of Electricity	RACPEUE
Private Gas Power Plant CO2 Emission	PSGPPCE	Total Current Cost of Generating Electricity Consumed by Consumer Sectors	TCCGECCS
Large Industries Gas Power Plant CO2 Emission	LIGPPCE	Current Average Cost of Producing Each Unit of Electricity	CACPEUE
Ministry of Power Gas Power Plant CO2 Emission	MOPGPPCE	Subsidy Allocated to Domestic Electricity According to the Current Production Cost	SADEACPC
		Domestic Electricity Sale Income	DESI
Ministry of Power CO2 Emission of Electricity Generation	MOPCEEG	Subsidy Allocated to Domestic Electricity According to the Real Cost of Production	SADEARCP
Ministry of Power Combined Cycle Power Plant CO2 Emission	MOPCCPPCE	Share of Renewable Power Plants in Gross Power Generation	SRPPGPG
Disel Power Plant CO2 Emission	DPPCE	Current Production Cost of Each Unit Thermal Electricity	CPCEUTE
Ministry of Power Steam Power Plant CO2 Emission	MOPSPPCE	Production Cost of Each Unit Renewable Electricity	PCEURE
Ministry of Power CO2 Emission Damage of Electricity Generation	MOPCEDEG	Steam Power Plant Nominal Capacity	SPPNC
Damage of Per Unit CO2 Emission	DPUCE	Steam Power Plant Nominal Capacity Increase	SPPNCI
Thermal Power Plant CO2 Emission	TPPCE	Steam Power Plant Exhaustion Rates	SPPER
Combined Cycle Power Plant CO2 Emission	CCPPCE	Steam Power Plant Useful Life	SPPUL
Steam Power Plant CO2 Emission	SPPCE	Steam Power Plant Capacity Increase Rate	SPPCIR
Total Damage of Thermal Power Plant CO2 Emission	TDTPPCE	Steam Power Plant Maintenance Rates	SPPMR
Export	E	Steam Power Plant Capacity Development	SPPCD
Import	I	Steam Power Plant Exhaustion Rates	SPPER
Export Ratio	ER	Steam Power Plant Maintenance Coefficient	SPPMC
Import Ratio	IR	Steam Power Plant Practical to nominal capacity Ratio	SPPPNCR
Electricity Losses and Power Plant Consumption	ELPPC	Steam Power Plant Practical Capacity	SPPPC
Electricity Losses and Power Plants Consumption Ratio	ELPPCR	Steam Power Plant Practical capacity to Gross Power Generation Ratio	SPPPCGPGR
Electricity Shortage/Surplus	ESS	Steam Power Plant Gross Power Generation	SPPGPG
Required Import	RI	Steam Power Plant Alocated Income to Development	SPPAID
Electricity Surplus	ES	Thermal Power Plant Alocated Income to Development	TPPAID
Export CO2 Damage	ECD	Steam Power Plant Allocation Income Coefficient	SPPAIC
Alocated Income to Capacity Development According to Import and CO2 Emission cost	AICDAICEC	Steam Power Plant Annual Cost of Capacity Development	SPPACCD
Export to Consumption Ratio	ECR	Steam Power Plant Development Time	SPPDT
Total Import Cost	TIC	Total Thermal Gross Power Generation	TTGPG
Alocated Income to Capacity Development	AICD	Combined Cycle Power Plant Gross Power Generation	CCPPGPG
Export Income	EI	Disel Power Plant Gross Power Generation	DPPGPG
Coefficient of Allocating Export Income to Renewable Capacity Development	CAEIRCD	Gas Power Plant Gross Power Generation	GPPGPG

**Table:** 

Equation	R^2	F	(1)
*HCPEnC=*166.104−(8.11519e+06/Pop)	0.874	110.8	(2)
$AEnC=1.574*\mathrm{Exp}\left(\left(6.837*\hat{10}-7\right)*GDP\right)$	0.891	130.2	(3)
ITEnC=1.78*(10^-7)*(GDP 0^1.371)	0.934	225.5	(4)
CEP=(Exp((−251.9)+(0.129*Time)))	0.933	236.6	(5)
HEP=(3.3*10^(-117))*((1.146)^Time)	0.932	234.25	(6)
*AEP=*Exp(-405.4+(0.204*Time))	0.918	189.4	(7)
ITEP=(2.76*(10^-93))*Exp(0.109*Time)	0.943	266.09	(8)
*GDP = INTEG (GDPI, 1.27919e+06)*	(9)		
*Pop= INTEG (PopI,65301.3)*	(10)		
AEC=(AECS/100)*CF*AEnC	(11)		
TEC=SLC+ITEC+AEC+CEC+HEC+PEC	(12)		
TREGCCS=((ITEC+AEC+CEC+HEC)*RACPEUE)	(13)		
TCCGECCS=((ITEC+AEC+CEC+HEC)*CACPEUE)	(14)		
SADEACPC=IFTHENELSE((TCCGECCS−DESI)<0,0,TCCGECCS−DESI)	(15)		
SADEARCP=IFTHENELSE((TREGCCS−DESI)<0,0,TREGCCS−DESI)	(16)		
CACPEUE=(((1−SRPPGPG)*CPCEUTE)+(SRPPGPG*PCEURE))	(17)		
*SLC=4117+RAMP(300, 2002, 2005)-RAMP(250, 2005,2011) + RAMP(210,2011,2018)*	(18)		

**Table:** 

*SPPNC= INTEG (SPPNCI-SPPER,14776)*	(19)
*SPPUL= 25*	(20)
SPPNCI=(SPPNC*SPPCIR) + SPPMR + SPPCD	(21)
SPPMR=SPPER*SPPMC	(22)
*SPPER=SPPNC/SPPUL*	(23)
*SPPPNCR=0.97-RAMP(0.03,2014,2015)-RAMP(0.027,2017,2018)*	(24)
*SPPPC*=SPPNC*SPPPNCR	(25)
*SPPPCGPGR=0.17-STEP(0.007, 2004) + STEP(0.009,2012)*	(26)
*SPPGPG=SPPPC/SPPPCGPGR*	(27)
SPPAID= TPPAID*SPPAIC	(28)
*SPPACCD=192.6*	(29)
*SPPCD=DELAY FIXED(IF THEN ELSE(Time<2021, 0, SPPAID/SPPACCD), SPPDT, 0)*	(30)
*TTGPG=CCPPGPG + SPPGPG + DPPGPG + GPPGPG*	(31)
*TPPAS=TPPTRGC-TPPTCGC*	(32)
*TRGPG=WPPGPG + HRPPGPG + HPPGPG + BPPGPG + SOPPGPG + NPPGPG*	(33)
RPPTGC= PCEURE*TRGPG	(34)
*TGPG=TTGPG + TRGPG*	(35)

**Table:** 

*MOPSGPPGG=0.9776-RAMP(0.076,2005,2010)-RAMP(0.03,2010,2013)-RAMP(0.11,2013,2015)*	(36)
MOPGPPGG= GPPGPG*MOPSGPPGG	(37)
LIGPPGG= GPPGPG*LISGPPGG	(38)
PSGPPGG=GPPGPG*PSSGPPGG	(39)
*PSIGPPCE=0+RAMP(787.06,2005,2006) + RAMP(211.6,2007,2008)-STEP(249.4,2009) + STEP(108,2010)-RAMP(340,2013,2014)-RAMP(16,2014,2017) + RAMP(40,2017,2019)*	(40)
MOPGPPCE= MOPGPPGG*MOPIGPPCE	(41)
LIGPPCE=LIGPPGG*LIIGPPCE	(42)
PSGPPCE=PSGPPGG*PSIGPPCE	(43)
*GPPCE=PSGPPCE + LIGPPCE + MOPGPPCE*	(44)
*MOPCEEG=(MOPCCPPCE + DPPCE + MOPGPPCE + MOPSPPCE)*	(45)
MOPCEDEG=(MOPCEEG/1e+06)*DPUCE	(46)
*TPPCE= (DPPCE + CCPPCE + SPPCE + GPPCE)/1e+06*	(47)
TDTPPCE=TPPCE*DPUCE	(48)

**Table:** 

E=ER*TGPG	(49)
*I*=IR*TEC	(50)
ELPPC= ELPPCR*TGPG	(51)
*ESS=TGPG + I-TEC-E-ELPPC*	(52)
*RI*=*IF THEN ELSE(Time<2020, 0, IF THEN ELSE(ESS<0, abs(ESS), 0))*	(53)
*ES= IF THEN ELSE(Time<2022, 0, IF THEN ELSE(ESS>0, ESS, 0))*	(54)
ECD=TDTPPCE*ER	(55)
*AICDAICEC=IF THEN ELSE(Time<2022, 0,* min(EI,((ECR*TIC) + ECD	(56)
AICD=(EI*CAEIRCD) + AICDAICEC	(57)

## Appendices

### B) Equations: Consumption subsystem equations

Production subsystem equations.

Emission subsystem.

Trade subsystem.
